# Supplementary figures and images for: The ASC Speck and NLRP3 Inflammasome Function Are Spatially and Temporally Distinct
Source: Front Immunol. 2021 Oct 21;12:752482. doi: 10.3389/fimmu.2021.752482 (PMC8566762; doi:10.3389/fimmu.2021.752482)

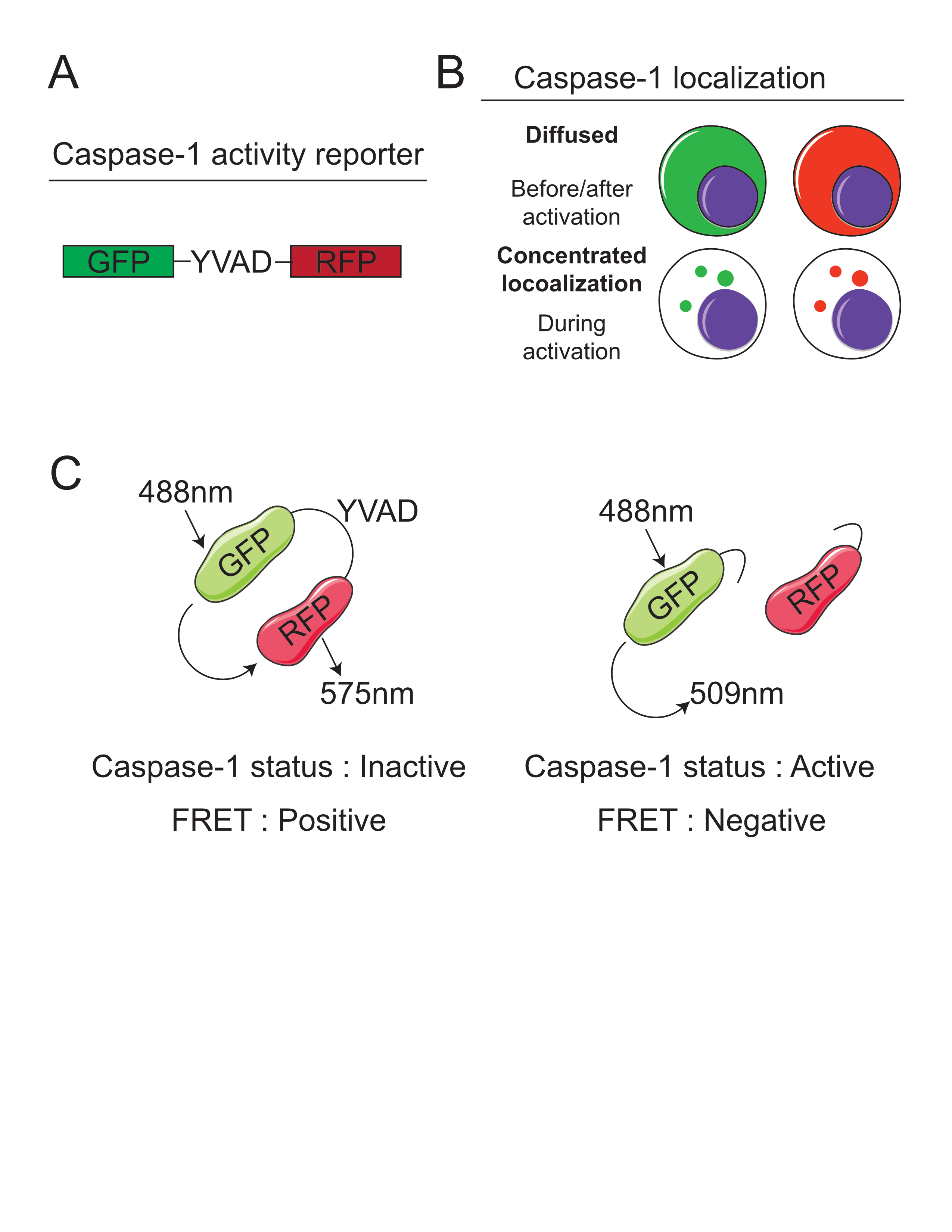

Supplement: Supplementary Figure S1 — Schematic representation of FRET based bi-fluorescent caspase-1 activity reporter. (A) Schematic representation of bi-fluorescent reporter for caspase-1 activity. GFP and RFP are linked by the caspase-1-specific recognition amino acid sequence “YVAD”, which is also present in pro-IL-1β. (B) The expected distribution patterns of caspase-1 reporter before, during, and after caspase-1 activation. (C) Schematic showing the cleavage states of the bi-fluorescent reporter allowing FRET-quantitation of cleavage by caspase-1. [file Image_1.tiff]

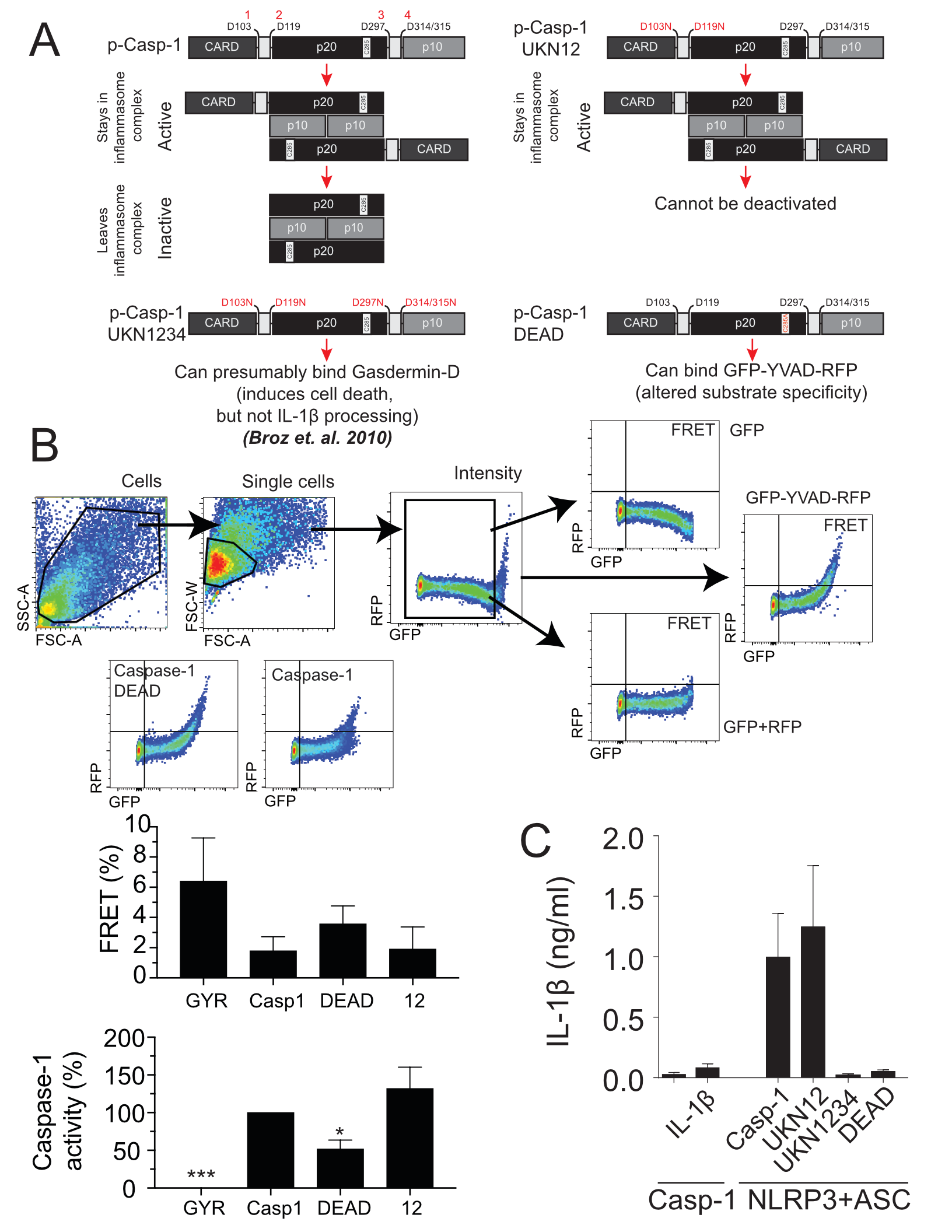

Supplement: Supplementary Figure S2 — Schematic representation of Caspase-1 mutants, their physiological phenotype and a FRET based bi-fluorescent caspase-1 activity reporter. HEK293T cells were transfected with 100 ng Flag-NLRP3, 100 ng myc-ASC,400 ng IL1B (B) or GFP-YVAD (D), 400 ng CASP1 (WT or mutants) or empty vector (EV). (A) Schematic representation of caspase-1 mutants and their expected phenotypes. The position of five aspartic acid residues are shown (103, 119, 297, 314 and 315). Aspartic acids 103 and 119 are the cleavage sites between the CARD domain and p20 subunit of caspase-1. Aspartic acid residues 297 and 314/315 are the cleavage sites between the p20 and p10 subunit of caspase-1. The caspase-1 UKN12 mutant (D103N/D119N) can be activated but cannot be released or deactivated. All the aspartic acid cleavage sites have in the caspase-1 UKN1234 mutant have D→N mutations, making this mutant non-activatable. The caspase-1 DEAD mutant has the caspase deactivating C285A active site mutation. (B) The gating strategy to determine caspase-1 activation by flow cytometry. (From left to right: Top) The SSC-A vs FSC-A plot allows to eliminate debris and gate for cells. The FSC-W vs FSC-A plot allows to gate for single cells. The singlets were then plotted for optimum GFP intensity (PE-A vs FITC-A). The optimum GFP intensity cells were then plotted for FRET and a quadrant gate was made to determine FRET positive cells. Representative plots showing the absence of events in FRET channel for cells transfected with GFP-alone or GFP and RFP expressed on different plasmids. Events in FRET channel can be recorded only in cells transfected with GFP-YVAD-RFP. (Bottom) The quadrant plot showing FRET intensity of GFP-YVAD-RFP (bi-fluorescent reporter) in FRET channel of samples transfected with caspase-1 or caspase-1 DEAD. Bar graphs showing % FRET positive cells and % caspase-1 activity (Calculations in Methods and Materials) (C). 24 hours post-transfection, culture supernatant was collected and IL-1β was measu [file Image_2.tiff]

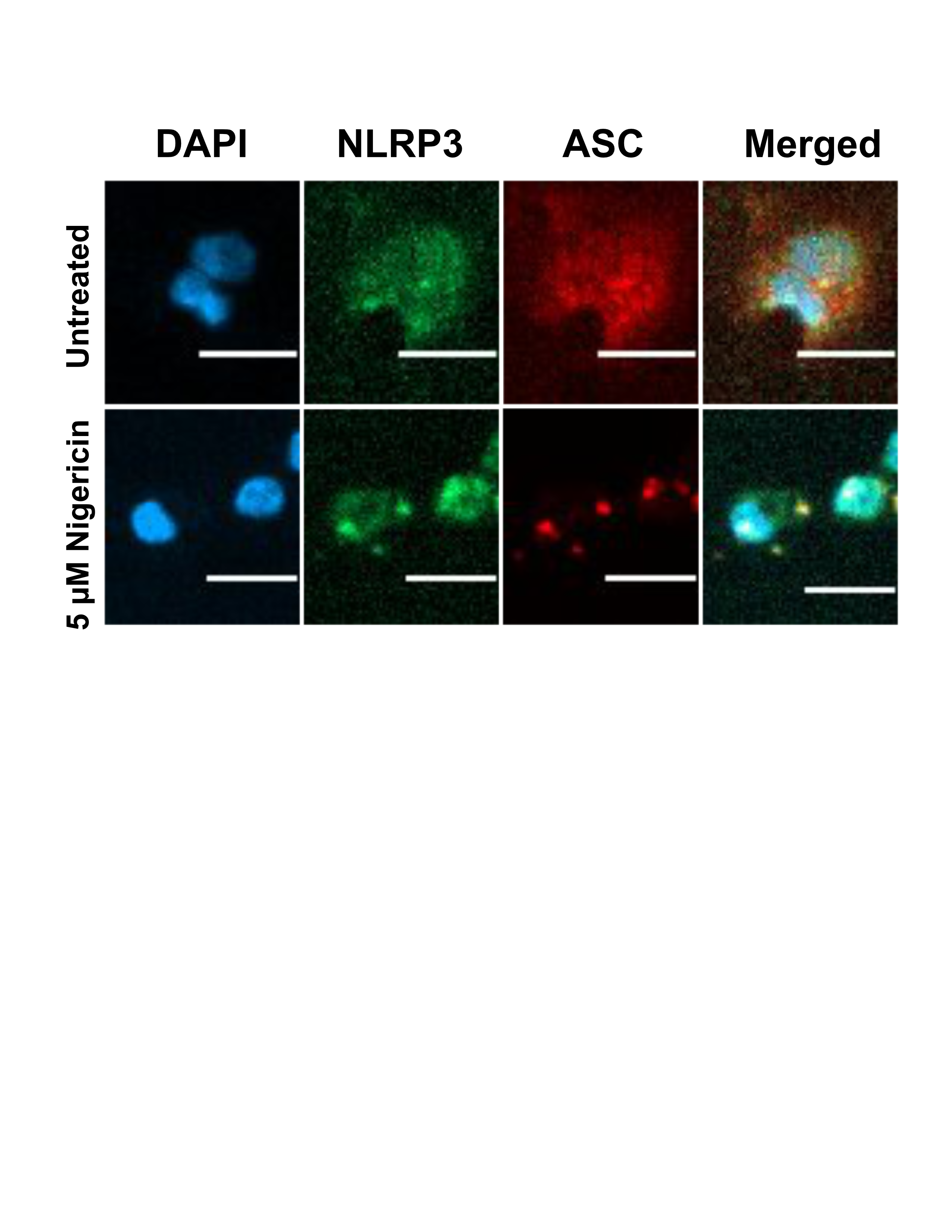

Supplement: Supplementary file 3 [file Image_3.tiff]

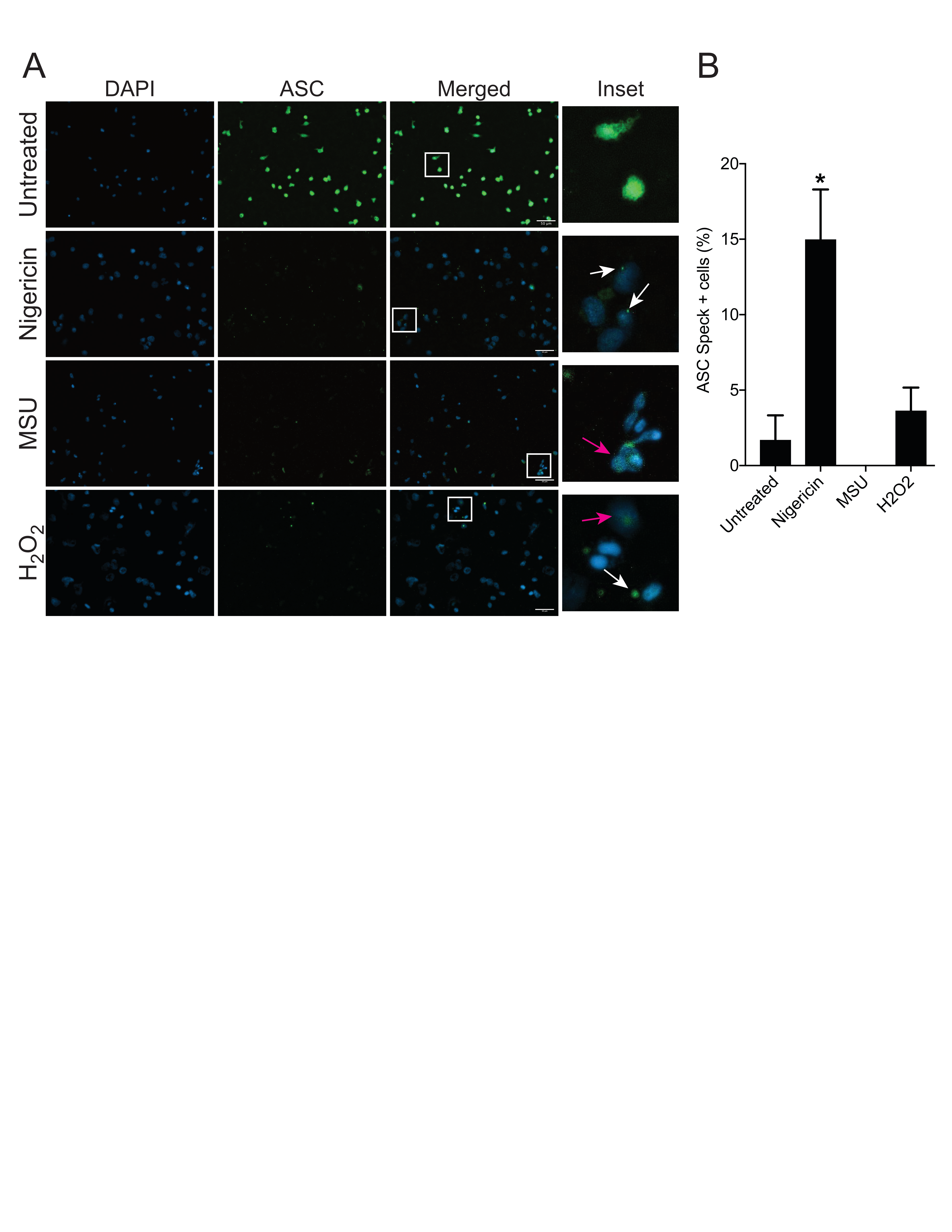

Supplement: Supplementary Figure S4 — NLRP3 inflammasome activation does not necessitate ASC speck formation. (A) THP-1 cells were primed with LPS for 4 hours and stimulated with the stated agonist (See Methods) Cells were fixed and permeabilized. Cells were stained for ASC and nuclei were stained with DAPI. Cells were analyzed for presence of specks. Scale bar = 50 µm. White arrows denote specks, yellow arrowhead indicates extracellular specks and red arrow indicates examples of non-speck cells. (B) Percentage of cells with intracellular specks and extracellular specks from A. Minimum of 100 cells from 3 field of views were analyzed. Mean percentages ± SEM are shown; **p < 0.01 & *p < 0.5, for comparison with untreated sample, one-way ANOVA followed by Dunnett’s multiple comparison tests. [file Image_4.tiff]

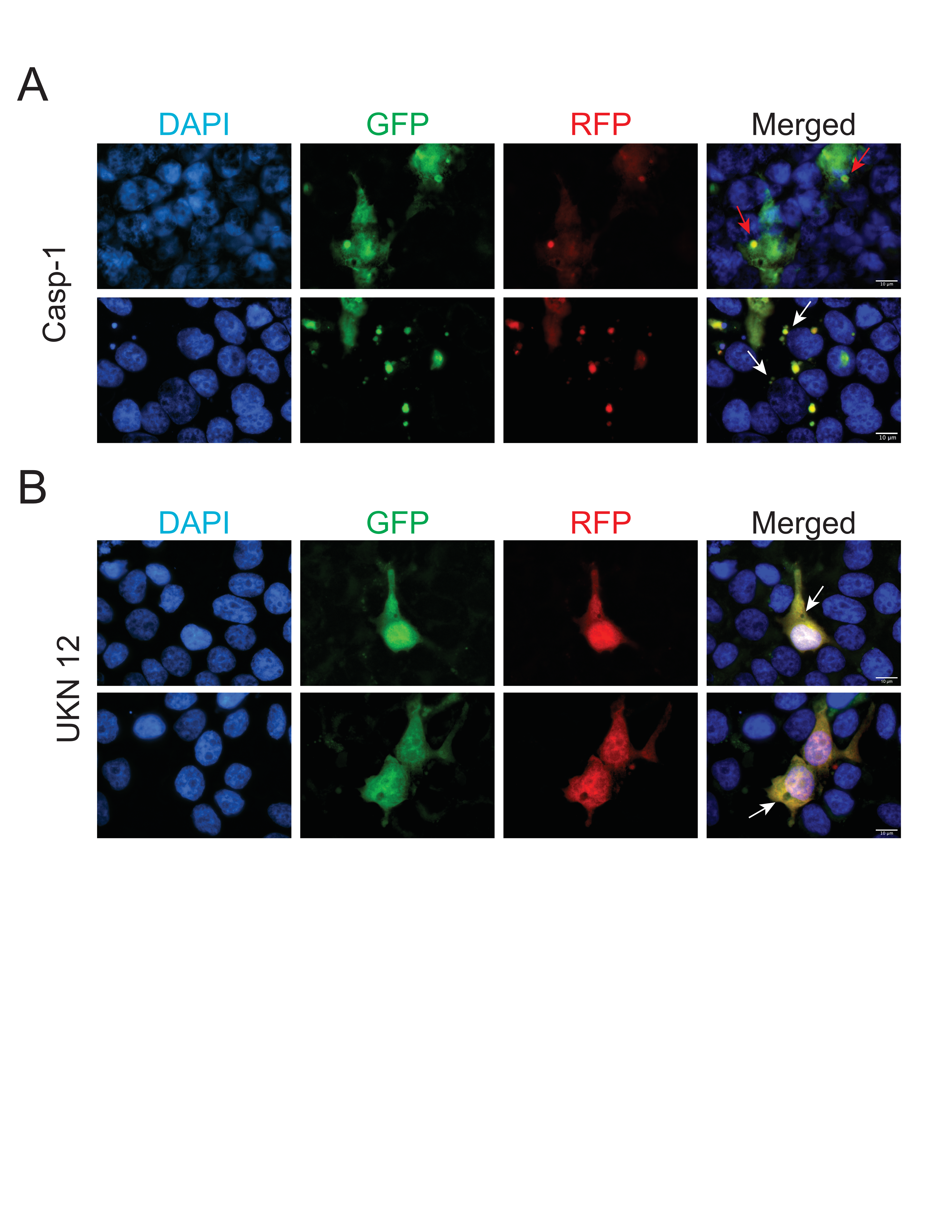

Supplement: Supplementary Figure S5 — Bi-fluorescent reporter recapitulates FLICA-like staining of active caspase-1. HEK293T cells were transfected with 100 ng myc-ASC, 100 ng NLRP3, 400 ng CASP1 (WT or UKN 12 mutant) and 400 ng GFP-YVAD-RFP. 18 hours post-transfection, cells were fixed, stained with DAPI and analyzed for GFP-RFP localization by microscopy. Colocalization (yellow) of GFP (green) and RFP (red) indicate binding of GFP-YVAD-RFP to active caspase-1 and thus reflects the cellular distribution of active caspase-1 sites. (A) (Top) Speck-like reporter fluorescence in some cells (red arrows; ~48% of cells, ). (Bottom) Multiple reporter aggregates in some cells (white arrows; ~25% of cells, ). (B) Two representative micrographs (top and bottom) showing a reporter-free region close to nucleus (white arrow; expected site of the speck; observable only with diffuse staining). Images are representative of three independent experiments. [file Image_5.tiff]

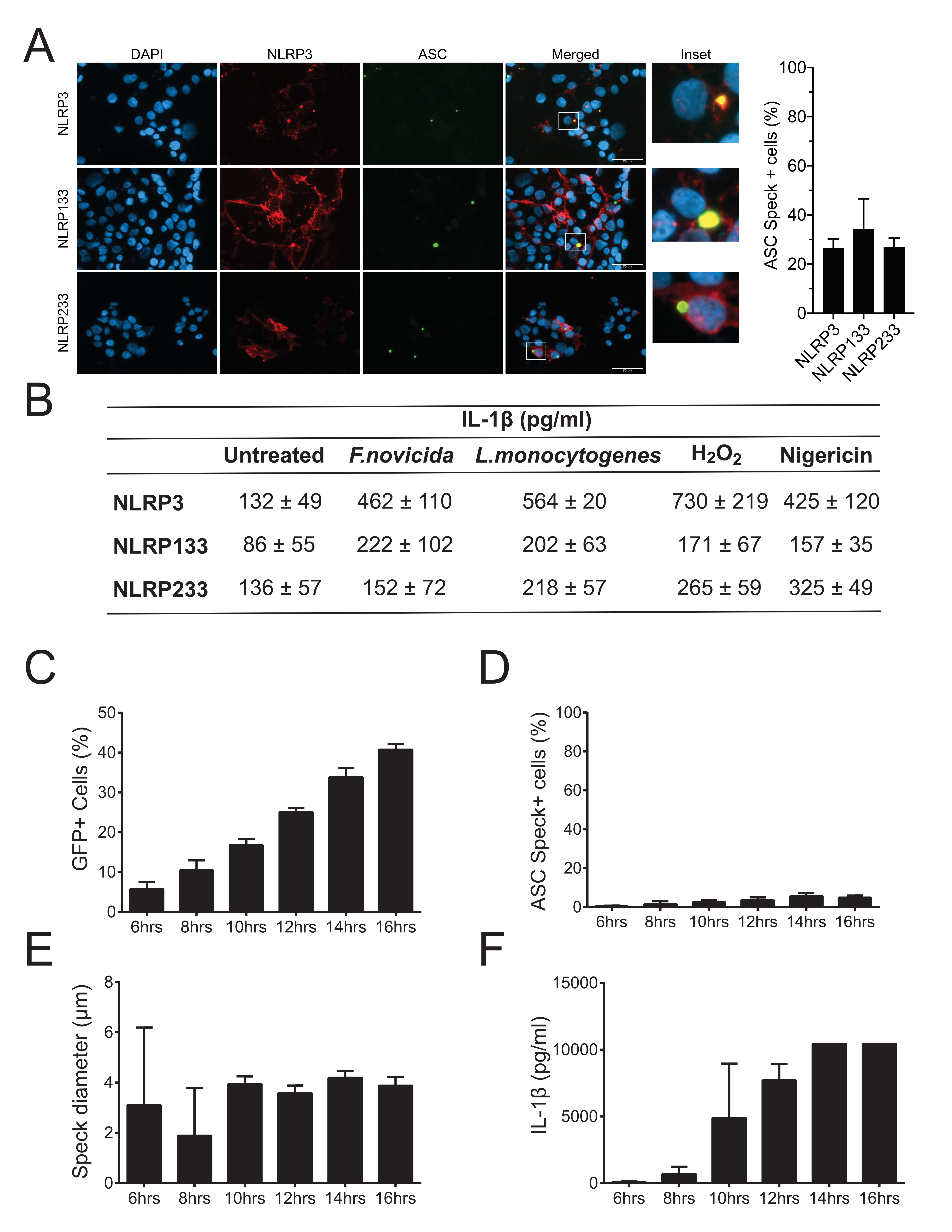

Supplement: Supplementary Figure S6 — Uncoupling of speck formation and IL-1β processing. (A) HEK293T cells were transfected with 1 µg myc-ASC and 1µg Flag-NLRP3 or a chimeric Flag-NLRP133 or Flag-NLRP233. After 18 hours cells, fixed and permeabilized cells were stained with anti-Flag (M2) and anti-ASC (Santa Cruz) and then analyzed for speck formation. Percentage of cells containing specks. Minimum of 140 cells were counted from 4 individual field of view; for NLRP233, 30 cells were counted. Data is presented as Mean ± SEM and analyzed using one-way ANOVA followed by Tukey’s multiple comparison test. (B) IL-1β processing in inflammasome reconstituted HEK293Ts expressing NLRP3 or the indicated chimeras and treated/infected with stated NLRP3 agonists (See Methods). (C-F) HEK293T cells were transfected with 100 ng NLRP3, 100 ng GFP-ASC, 20 ng CASP1 and 200 ng IL1B allowing agonist-independent maturation and release of active IL-1β. At stipulated time-points, cells and culture supernatants were harvested. Cells were fixed and analyzed for GFP expression (C), Culture supernatant was analyzed for released IL-1β (D), ASC speck (E) and speck diameter (F). [file Image_6.tiff]
